# The International Conference on Intelligent Biology and Medicine (ICIBM) 2016: putting systems biology to work

**DOI:** 10.1186/s12918-017-0461-x

**Published:** 2017-10-03

**Authors:** W. Jim Zheng, Jianhua Ruan, Hua Xu, Zhongming Zhao, Zhangdong Liu

**Affiliations:** 1 School of Biomedical Informatics, University of Texas Health Science Center at Houston, Houston, TX 77030 USA; 2 Department of Computer Science, The University of Texas at San Antonio, San Antonio, TX 78249 USA; 3 Center for Precision Health, School of Biomedical Informatics, The University of Texas Health Science Center at Houston, Houston, TX 77030 USA; 4 Human Genetics Center, School of Public Health, The University of Texas Health Science Center at Houston, Houston, TX 77030 USA; 5 The Jan and Dan Duncan Neurological Research Institute, Baylor College of Medicine, Houston, TX 77030 USA

## Abstract

Between December 8–10, 2016, the International Conference on Intelligent Biology and Medicine (ICIBM 2016) was held in Houston, Texas, USA. The conference included eight scientific sessions, four tutorials, one poster session, four highlighted talks and four keynotes that covered topics in 3D genome structure analysis and visualization, next generation sequencing analysis, computational drug discovery, medical informatics, cancer genomics and systems biology. Systems biology has been a main theme in ICIBM 2016, with exciting advances were presented in many areas of systems biology. Here, we selected seven high quality papers to be published in BMC Systems Biology.

## Introduction

For the past four years, ICIBM meeting has been covering extensive cutting edge research topics in systems biology. This year’s meeting is no exception. As the systems biology research advances, research focus has been shifted from methodology development to the application of systems biology approach to solve biomedical problems. We selected seven high quality papers from ICIBM 2016 meeting to reflect this trend. Most notably, three of these papers present various cutting edge researches in drug related topics, from network-based approaches for personalized drug repositioning, to cancer drug sensitivity prediction, and to repurposing FDA approved anticancer drugs. Other equally high quality works include modeling and hypothesis generation and transcription regulation modulated by alternative splicing. Even more impressive is the trend to apply systems biology approach for personalized therapy, e.g. employing patient-specific signaling network to predict effective drugs and reveal potential drug mechanisms. These works provide some novel insights in cutting edge systems biology research and their potentials in clinical applications.

## The science program for the ICIBM 2016 systems biology track

In the first paper by Wu et al. [1], a computational method, Mechanism and Drug Miner (MD-Miner), was developed by using a network-based computational approach to predict potential effective drugs. Different from widely-used drug repositioning approaches based on reverse gene signature, MD-Miner predicts potential effective drugs by comparing constructed disease signaling network of patients and individual drugs, which is helpful to reveal potential mechanisms of repositioned drugs action at the level of the signaling network. Worth noting is the use of patient specific signaling network from patient-derived gene expression profiles—a great potential for the true individualized therapy. The method was evaluated on the PC-3 prostate cancer cell line to show that it significantly improved the success rate of discovering effective drugs compared with the random selection, and could provide insight into potential mechanisms of drug action.

As drug effectiveness was such an important topic, it is not a surprise that the second selected paper by Turki and Wei [2] also developed a novel approach for cancer drug sensitivity prediction. In this work, cancer drug sensitivity was modeled as a linked prediction. The two proposed algorithms can overcome the issues of existing methods in dealing with poor quality of cell lines used in drug screening and the failure to adopt a new feature representation. This superiority over existing method was demonstrated by the results from method evaluation using Clinical trial data.

While the first two papers have very specific focus on algorithms for in-depth analysis and prediction of drug effectiveness and sensitivity, the third paper by Sun et al. [3] provided a broad survey of 150 FDA-approved anticancer drugs. The comprehensive analysis divided these drugs into 61 cytotoxic-based and 89 target-based drugs based on drug mechanism of action. Through building a cancer-drug network, this work found that cytotoxic drugs tend to be used to treat more cancer types than targeted drugs. A separate cancer-drug-target network was built from 89 targeted drugs to identify 133 novel drug-cancer associations, demonstrating the ability of the approach to find potential use of existing drugs for drug repurposing.

The role of alternative splicing in transcription regulation was studied by Li et al. [4] in the next selected paper. They constructed a regression-based linear model to infer whether the alternative splicing events of modulator proteins can affect the ability of key transcription factors in regulating the expression levels of their transcriptional targets. Using Kidney Renal Clear Cell Carcinoma (KIRC) RNA-Seq data downloaded from the Cancer Genome Atlas (TCGA), the developed model identified 828 modulation relationships between the splicing levels of modulator proteins and activity levels of transcription factors. The analysis suggests, for the first time, that exon inclusion levels of certain regulatory proteins can affect the activities of many transcription factors, providing a potential novel mechanism of how splicing variation impacts the cellular function and dysregulation of splicing outcome can lead to various diseases.

Cancer signature is the focus of the next selected paper. In this work, Yang et al. [5] incorporated genomic, transcriptomic and clinical data to reveal an embryonic stem cells (ESCs) like cancer signature in high-risk neuroblastoma (HR-NB), the most common extracranial solid tumor in children. The work identified two novel prognostic gene-set pairs with multi-scale oncogenic defects. By analyzing the different biological functions of the gene components of these signatures, the work provided some novel insights into the PRC2-mediated tumor cell growth and differentiation in neuroblastoma and could help to reduce relapse and mortality rates of cancer patients.

Research into cancer-stem cell link is also described by Li et al. [6]. In this work, functional alteration of hematopoietic stem cells (HSCs) in leukemic environment was studied through two genes, Maff and Egr3, that have opposite functions but both highly expressed in HSCs. Combining experimental approaches such as microarray, qRT-PCR and flow cytometry, HSCs were analyzed extensively to generate transcription profiles for kinetic modeling of cell cycles. Using Ordinary Differential Equation (ODEs), the actions of these two genes on cell cycle were modeled, suggesting a possible “adaptation – suppression” process in HSCs in leukemic environment. The system modeling approach provided novel insight that can guide future experimental investigation of leukemia-induced functional alterations of hematopoietic cells.

Computational modeling approach was also developed by Li et al. [7] to predict early stages of adult hippocampal neurogenesis in the next selected paper. In this work a branching process theory based computational model was developed to represent the early stage hippocampal neurogenic cascade. Combining experimental investigation with this stochastic model simulation, the overall efficiency of neurogenesis in both normal and disease conditions were analyzed to estimate important parameters such as the apoptotic rates at different stages of the neurogenic cascade. The developed model can be used to predict overall efficiency of hippocampal neurogenesis in both normal and diseased conditions, as well as simulating the behavior of the neurogenic system under perturbations.

## References

[1] Wu H, Miller E, Wijegunawardana D, Regan K, Payne P, Li F (2017). MD-miner: a network-based approach for personalized drug repositioning. BMC Syst Biol.

[2] Turki T, Wei Z (2017). A link prediction approach to cancer drug sensitivity prediction. BMC Syst Biol.

[3] Sun J, Wei Q, Zhou Y, Wang J, Liu Q, Xu H (2017). A systematic analysis of FDA-approved anticancer drugs. BMC Syst Biol.

[4] Li J, Wang Y, Rao X, Wang Y, Feng W, Liu Y (2017). Roles of alternative splicing in modulating transcriptional regulatio. BMC Syst Biol.

[5] Yang XH, Tang F, Shin J, Cunningham JM (2017). Incorporating genomic, transcriptomic and clinical data: a prognostic and stem cell-like MYC and PRC imbalance in high-risk neuroblastom. BMC Syst Biol.

[6] Li R, Wang Y, Cheng H, Liu G, Cheng T, Liu Y, Liu L (2017). System modeling reveals the molecular mechanisms of HSC cell cyclealteration mediated by Maff and Egr3under leukemia. BMC Syst Biol.

[7] Li B, Sierra A, Deudero JJ, Semerci F, Laitman A, Kimmel M, Maletic-Savatic M (2017). Multitype Bellman-Harris branching model providesbiological predictors of early stages of adult hippocampalneurogenesis. BMC Syst Biol.

